# Computational Biology Resources Lack Persistence and Usability

**DOI:** 10.1371/journal.pcbi.1000136

**Published:** 2008-07-18

**Authors:** Stella Veretnik, J. Lynn Fink, Philip E. Bourne

**Affiliations:** 1 San Diego Supercomputer Center, University of California San Diego, La Jolla, California, United States of America; 2 Skaggs School of Pharmacy and Pharmaceutical Sciences, University of California San Diego, La Jolla, California, United States of America; Millennium Pharmaceuticals, United States of America

Innovation in computational biology research is predicated on the availability of published methods and computational resources. These resources facilitate the generation of new hypotheses and observations both on the part of the creators and the scientists who use them. These methods and resources include Web servers, databases, and software, both complex and simple, that implement a specific procedure or algorithm. Usually, a resource is maintained by the laboratory in which it was initially developed. We would assert that there is a growing level of frustration among scientists who attempt to use many of these resources and find that they no longer exist or are not properly maintained. Whether you agree or disagree with this statement and the evidence that follows, we welcome your thoughts and invite you to add a Comment to this article to share your own experiences and perspectives.

It is timely to visit this situation in more detail. The International Society for Computational Biology (ISCB) is reviewing its position on software sharing, and this journal is now doing the same (the views expressed here are not necessarily those of the journal—this is a personal perspective and not an editorial). To help us gain a better understanding of the resource situation, we took on two simple experiments: first, a review of the persistence of Web servers, and, second, an experience creating a metaserver—a Web site where users can come and run a variety of methods to compare results. Here is what we found.

## Web Server Persistence

**Figure 1:**
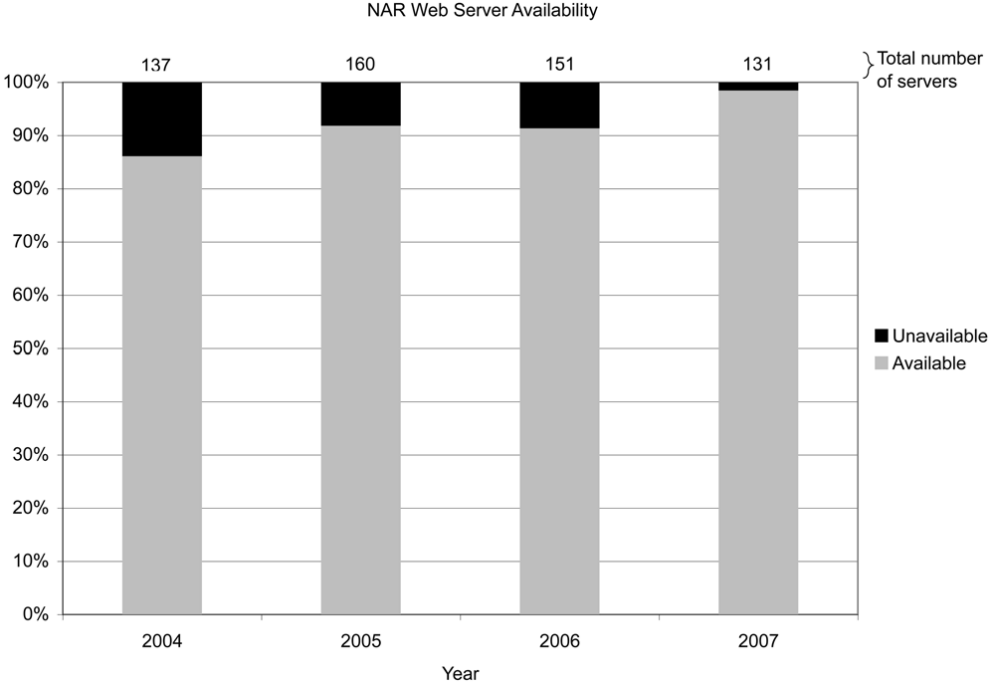
NAR Web server availability.

To evaluate the persistence of biology Web servers, we extracted all the URLs from the Nucleic Acids Research (NAR) Web server issues over the past four years since its inception (Figure 1). We then ran a simple script to determine which status code was returned when each URL was visited. Web servers were said to exist if a status code of 200, 301, or 302 was returned. If an error-type status code was returned (400, 401, 403, 404, 405, 406, 408, 411, 500, 501, 503), the URL was manually checked.

Of those servers published four years ago, 14% no longer appear to be active, and a significant number of those published two and three years ago were similarly unavailable. One can speculate as to the reasons for these findings. The sites may have been down when we tried to access them but are available most of the time (although URLs with an error status were visited twice, a week apart). More likely, they are no longer maintained either because of a lack of funding to support maintenance, the responsible parties have left the laboratory, or there simply has been a shift in emphasis by the laboratory and/or the community. For example, a newer resource may provide a more recent and well-accepted development. Given that journals describing these resources are more dynamic than they used to be, for example in adding commentary to a paper, these findings beg the question: Should journals report when the resource appears to become unavailable? This might encourage authors to keep the server operating or to not publish the resource at all if they are not inclined to maintain it. Your thoughts on this would be welcome.

## Software Availability

It is difficult to acquire quantitative information describing software availability. Rather than attempting to perform a comprehensive survey, we describe a specific experience from our own laboratory.

Decomposition of protein structures into domains is one of the oldest and still active areas of research in computational biology. As soon as a few dozen structures were solved, methods for partitioning these structures into compact, globular units (coined domains) appeared. The complexity and sophistication of the methods increased as more structures and more powerful computers became available, and this trend still continues today. More than 30 methods have been published in the last 30 or so years, with two new methods being reported in 2007. This information might lead one to believe that there are now a large number of computational methods from which to choose. Our experience indicates otherwise.

Many of the methods published since 1995 are difficult or impossible to obtain. The description of each new method's publication points out how its method is better than the previous ones. However, it is hard to objectively evaluate these claims because the software is simply unavailable. In addition, new methods are often tested using different sets of data than previously published methods, so a direct quantitative comparison cannot be made.

In our attempt to analyze and benchmark methods for domain assignments, we are continuously working (or attempting to work) with the authors of the methods. With few exceptions, the software is not submitted along with article. When we contact the authors to obtain the software implementation of their published method and to run that software locally, one of the following scenarios takes place (ranked from worst to best).

1. The authors of the paper do not respond. Authors rarely respond after the first request, but some do not respond no matter how many times we contact them.
2. Authors of the software engage in a dialog and promise to provide the software. In the end, however, they never provide the code due to lack of time, resources, and (we suspect) lack of incentive.
3. The student or postdoctoral fellow who wrote the software has since left the group, and the author, who has started new projects, has no time or interest in providing the software. Sometimes, it is also revealed that the software requires subjective manual post-processing, and it could thus be argued that the published results are irreproducible.
4. The software is eventually provided, but it cannot be made to run, and the authors are not eager to help.
5. Authors say they need to work further on the software, and eventually (over months or years) we receive it, it works, and if we have problems authors continue to collaborate with us.
6. The authors provide their software immediately, assist us with our local installation, and even improve usability of their software upon our suggestions.

Of 14 methods we attempted to obtain, some over a period of four years, we gained access to six methods (covering the period from 1994 to 2008) that we can run locally or remotely on a consistent basis.

## Toward a Solution

Based on our experience, it can be said that the notion of what constitutes “software” in the field of computational biology is variable. Many programs/Websites are developed as part of a graduate student's thesis without any forethought given for their future maintenance. Should these be published in peer-reviewed journals and presented as legitimate resources to the scientific community? Certainly there is pressure to do so as part of the academic process. But in the longer term are we doing the scientific community a disservice?

Currently, there is little incentive to encourage a responsible approach to software/Website support. Extreme demands on scientists' time and the constant push toward novel research make it difficult to maintain existing resources. Funding agencies seem willing to fund new developments of resources but not their ongoing maintenance, unless they are known to be vital to the field at large. Journals may or may not review resources described in the submitted paper, and there does not seem to be a business model accepted by the academic community by which these resources can be maintained. Open sourcing seems to be the most viable solution at this time.

The issue of resource persistence and usability would seem to be of particular importance to the field of computational biology. This issue plays a significant role in how our discipline is perceived by the broader scientific community. As such, we suggest that, as a community and as individual scientists, we must be more vigilant and responsible when we publish new resources. It would seem no longer sufficient to write in a paper that “software is available from the authors upon request” or to publish a Web server that will quickly become obsolete.

By way of full disclosure, we (the authors) admit that we have all been guilty of poor software support and the creation of transient Web servers (although we have also all been helpful, at other times, with software support as well.) The important issue to consider is how all of us can do better in the future. We list a few possible scenarios that might improve the availability and usefulness of published computational methods and resources and invite your comment.

1. Authors should not be able to publish a method or relevant performance statistics without providing the software and tested datasets to the journal or to a stable, publicly available third-party repository. The goal here is to support reproducibility at any time subsequent to the paper being published. Furthermore, documented software should be submitted as part of the peer review process.
2. Authors, or at least the primary author, of the method, should sign an agreement at the time their paper is accepted for publication that they will actively maintain the software and make it available for a specified period.
3. Since authors of the methods are often graduate students or postdoctoral fellows, the head of the laboratory, as mentor, should insist on better standards of software practice and take responsibility for support and maintenance. Perhaps maintenance of the computational resources should be treated similarly to the author's publications, where status and level of use of each resource is reported as a prerequisite for publication. At the very least, the time the resource was last updated should be displayed prominently.
4. The computational biology research community (not any one journal) should develop standards for software and Web servers, including guidelines on adequate testing, documentation, and the provision of benchmark datasets. In addition, the community should mandate the deposition of software in a publicly available open source repository.

Such scenarios may be thwarted by institutional copyright issues that complicate deposition of software to open source or publicly available repositories. While a complex and contentious subject, it could be overcome in many cases by more insistent policies by funding agencies before the resource is developed and by scientists requesting from their institutions that the resources they develop be fully open prior to accepting funding. Certainly only a small percentage of the software currently in use by computational biologists is available from an open source archive. Beyond institutional and copyright issues, there are the scientists themselves who publish the work but do not want to go to the trouble of making the resource easy to use. Wouldn't it seem that evidence of usability through suitable documentation and accessibility should be prerequisite to publishing a paper when that paper is about such a resource?

What do you, as members of the computational biology research community, think, and what are you willing to do? Is our integrity being compromised by the resources we are making public? Is this concern overstated? Are there other approaches to solving the problem? Please post a Comment to this article to make your views known.

